# Blood pH, hemoglobin function and oxygen transport throughout the dive cycle of emperor penguins

**DOI:** 10.1242/jeb.251044

**Published:** 2025-11-19

**Authors:** Paul J. Ponganis, Cassondra L. Williams

**Affiliations:** ^1^ Center for Marine Biotechnology & Biomedicine, Scripps Institution of Oceanography, University of California San Diego, La Jolla, CA 92093-0204, USA; ^2^ National Marine Mammal Foundation, 2240 Shelter Island Drive, San Diego, CA 92106, USA

**Keywords:** Arterial, Carbon dioxide, Hemoglobin saturation, Oxygen, pH, Venous

## Abstract

Optimal blood O_2_ transport during a dive and during the apnea-to-eupnea transition at the surface is essential as cardiovascular adjustments and the onset of hyperventilation shift an animal from a state of hypoxia during late ascent to one of O_2_ replenishment and metabolic restoration at the surface. Because pH affects the O_2_ affinity of hemoglobin (Hb), we examined the relationship of blood pH to carbon dioxide and lactate in emperor penguins (*Aptenodytes forsteri*), and determined that pH 7.3 was an appropriate lower bound for blood pH during the dive and surface intervals. Hb saturation calculated from *P*_O_2__ profiles at pH 7.5 to 7.3 revealed that (a) arterial oxygenation remained high regardless of pH until late ascent; (b) the blood O_2_ store depletion rate increased by 27% with an end-dive pH of 7.3 versus 7.4, resulting in a 5% increase in the total body O_2_ store depletion rate; the blood O_2_ store was not depleted even at dive durations 2 times the aerobic dive limit; and (c) restoration of blood O_2_ in the surface period was faster at higher pH, but occurred within 3 min regardless of pH. We conclude that (i) despite elevated blood O_2_ store depletion rates at a final pH of 7.3, the change in total body O_2_ store depletion rate was small, and (ii) blood pH and the high affinity and Bohr effect of emperor penguin Hb were most critical during late ascent and the apnea-to-eupnea transition, the most physiologically challenging and dynamic phases of a dive.

## Teaser

**Summary:** Blood pH and the high affinity and Bohr effect of emperor penguin hemoglobin are most critical during the late ascent of dives and the apnea-to-eupnea transition.

## INTRODUCTION

Efficient foraging in diving penguins is determined by not only the ability to dive aerobically but also by the capacity to recover quickly and continue diving (Kooyman et al., 2021). While increased oxygen (O_2_) stores and the diving response (i.e. slow heart rates and vasoconstriction) allow for blood O_2_ conservation and longer aerobic dives, the ability to dive repetitively requires high heart rates and the onset of hyperventilation to shift an animal from a state of hypoxia during late ascent to one of O_2_ replenishment and metabolic restoration at the surface (Ponganis, 2015; Ponganis et al., 2011). In addition, blood pH changes throughout the dive cycle as a result of the buildup of carbon dioxide (CO_2_) during the dive and the additional washout of tissue CO_2_ and sometimes lactate during the transition from apnea to eupnea (Kooyman et al., 2021; Qvist et al., 1986; Scholander and Irving, 1941). Despite these changing conditions, optimal O_2_ transport and hemoglobin (Hb) function are required throughout both these distinct physiological states.

Changes in blood pH affect O_2_ transport and Hb function via changes in the O_2_ affinity of Hb (Hb–O_2_ affinity) (Signore et al., 2021). However, the effects of these pH changes on O_2_ transport during dives have not been examined in penguins for several reasons. First, there are few blood pH data available during dives of penguins (Ponganis et al., 2009). In prior estimates of blood O_2_ depletion and repletion in emperor penguins (*Aptenodytes forsteri*), we used a single end-of-dive pH that was assumed most representative over the wide range of dive durations in the study (Meir and Ponganis, 2009). Second, there are no estimates of lower limits of pH during dives or surface intervals of diving birds. As a result, the effects of changes in blood pH on blood O_2_ transport and depletion during dives or at the surface are unknown.

Therefore, here we examined the relationship of blood pH to the partial pressure of CO_2_ (*P*_CO_2__) and lactate concentration to determine an appropriate lower bound for blood pH during the dive and surface interval. We then used the O_2_–Hb dissociation curve at that pH to convert blood partial pressure of O_2_ (*P*_O_2__) profiles to Hb–O_2_ saturation profiles representative of that lower pH limit (Meir and Ponganis, 2009). With this approach, we refined our evaluation of the patterns and rates of blood O_2_ depletion/repletion both during the dive and the apnea-to-eupnea transition, and better defined how and when adaptations in emperor penguin Hb function were most critical.

The emperor penguin is an excellent model to investigate blood O_2_ transport, Hb function and the effect of blood pH during the dive and during the dynamic apnea-to-eupnea transition. The aerobic dive limit (ADL, dive duration associated with the onset of post-dive blood lactate accumulation, 5.6 min) has been documented with blood lactate measurements (Ponganis et al., 1997). Blood *P*_O_2__ profiles and O_2_–Hb dissociation curves at different pH have also been documented in emperor penguins (Meir and Ponganis, 2009; Ponganis et al., 2009). Although blood pH data during dives were limited to the first few minutes of dives (Ponganis et al., 2009), there are many other unpublished data, collected opportunistically, that could be used to examine the regulation of blood pH.

For the benefit of readers, we first provide a brief review of O_2_–Hb dissociation curves, *P*_50_ (*P*_O_2__ at 50% Hb saturation, an index of Hb–O_2_ affinity), the Bohr effect (the change in Hb–O_2_ affinity that occurs with changes in pH), effects of increased CO_2_ and lactate during the breath hold, effects of ambient pressure at depth on lung and arterial *P*_O_2__, and venous Hb saturation and the Bohr effect. We conclude with the goals of this study.

### O_2_–Hb dissociation curve and Hb–O_2_ affinity

**Fig. 1.**
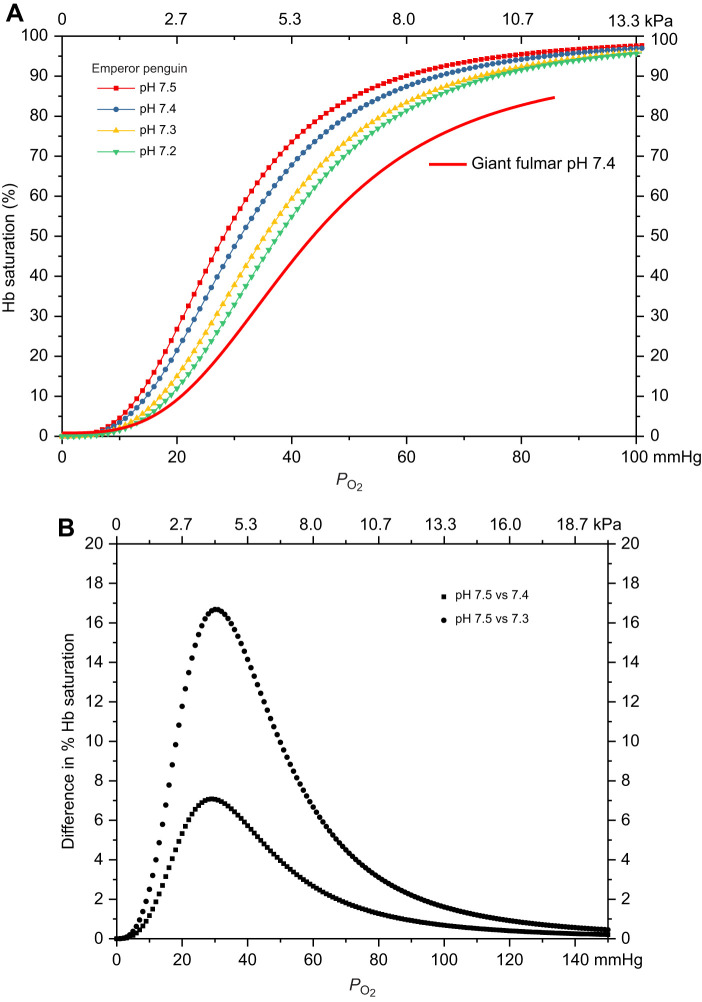
**Hemoglobin–oxygen affinity of emperor penguins.** The O_2_ affinity of emperor penguin hemoglobin (Hb) decreased with acidosis (the Bohr shift) but was still greater than that of the giant fulmar. (A) The *P*_50_ values [partial pressure of O_2_ (*P*_O_2__) at 50% Hb saturation] of emperor penguin Hb at pH 7.5, 7.4, 7.3 and 7.2 were 28, 31, 35 and 37 mmHg, respectively, while that of the giant fulmar was 44 mmHg (Meir and Ponganis, 2009; Milsom et al., 1973). (B) The difference in percentage Hb saturation due to the Bohr shift in the emperor penguin.

The O_2_–Hb dissociation curve illustrates the relationship between the amount of O_2_ bound to Hb and the blood *P*_O_2__ level (Signore et al., 2021; Storz, 2016). Hb's affinity for O_2_ and the sigmoidal shape of the O_2_–Hb dissociation curve determine the percentage of Hb molecules with O_2_ bound (Hb–O_2_ saturation, hereafter termed saturation) at a given *P*_O_2__. The increased Hb–O_2_ affinity of emperor penguin whole blood is exemplified by a decreased *P*_50_ and left-shifted dissociation curve in comparison to those in most birds (Fig. 1A). In the emperor penguin, the *P*_50_ ranged from 27 mmHg at pH 7.5 (pH at rest) to 37 mmHg at pH 7.2 (1 mmHg=0.133 kPa) (Meir and Ponganis, 2009). In contrast, one of the earliest studies to examine Hb function in marine birds found that the *P*_50_ of the giant fulmar (*Macronectes giganteus*) was 44 mmHg at pH 7.4 (Milsom et al., 1973). The emperor penguin Hb–O_2_ affinity resembles values in many mammals and in birds that live at high altitude (Lenfant et al., 1969; Meir and Ponganis, 2009; Milsom et al., 1973; Signore et al., 2021; Storz, 2016). A higher Hb–O_2_ affinity provides a higher saturation and blood O_2_ content at lower *P*_O_2__ values, allowing for greater hypoxic tolerance and greater utilization of the respiratory O_2_ store.

### The Bohr effect

However, as in other birds and animals, a decrease in pH (acidosis) results in the Bohr effect – a decreased Hb–O_2_ affinity and a shift in the dissociation curve to the right (Fig. 1A). As the O_2_–Hb dissociation curve shifts to the right, O_2_ is released more easily at higher *P*_O_2__ values, facilitating O_2_ transport to the tissues. Emperor penguins exhibit a large Bohr effect, meaning that a change in pH can significantly alter the affinity Hb has for O_2_ (Meir and Ponganis, 2009; Signore et al., 2021). The combination of a high Hb–O_2_ affinity and a large Bohr effect has long been considered to promote the uptake of O_2_ from the lungs as well as to promote delivery of O_2_ to tissues in penguins during dives and during the post-dive surface interval (Lenfant et al., 1969; Meir and Ponganis, 2009; Milsom et al., 1973; Signore et al., 2021).

### The effects of CO_2_ and lactate during and after dives

The response of blood pH to changes in CO_2_ level and lactate concentration ([lactate]) is dependent on the buffering capacity of blood and is especially critical to O_2_ transport during both the dive and the surface period because blood pH regulates Hb–O_2_ affinity via the Bohr effect. As CO_2_ in the blood builds up during the breath hold of a diving penguin, blood pH may drop, decreasing Hb–O_2_ affinity. This reduced Hb–O_2_ affinity enhances the release of O_2_ to tissues but may also impair O_2_ uptake from the lungs (Butler and Taylor, 1983; Hudson and Jones, 1986; Ponganis et al., 2009). Once eupnea begins, elevated cardiac output and tissue perfusion increase washout and transport of additional CO_2_ from tissues into veins, further reducing venous blood pH and O_2_ affinity. During this period, tachycardia and hyperventilation should enhance CO_2_ removal, which increases blood pH and the O_2_ affinity of Hb in pulmonary capillaries and the aorta, promoting O_2_ uptake from the lungs. For dives beyond the ADL, significant tissue washout of lactate into the blood typically begins during eupnea (Ponganis et al., 2009). In contrast to rapid CO_2_ removal in the lungs, a prolonged metabolic acidosis secondary to significant elevation of blood [lactate] after longer dives may lower Hb–O_2_ affinity and impair blood reoxygenation during the surface period.

### Hydrostatic pressure effects on lung *P*_O_2__ and arterial *P*_O_2__ and Hb saturation

In divers, the increase in ambient pressure at depth can result in a high *P*_O_2__ in the lung despite a decreased O_2_ fraction in the gas phase of the respiratory system. For example, if the respiratory percentage of O_2_ is 4%, respiratory *P*_O_2__ would be 29 mmHg at the surface but 120 mmHg at a depth of 30 m. Assuming lung *P*_O_2__ approximates arterial *P*_O_2__ values, the corresponding arterial Hb saturation should be high during much of the dive – until the bird nears the surface during late ascent (Stockard et al., 2005; Williams et al., 2021). Therefore, it is not surprising that arterial Hb saturation in emperor penguins remained high during most of the dive, not decreasing until late ascent (Meir and Ponganis, 2009). Furthermore, even in the presence of acidosis (i.e. 0.2 pH unit decrease from baseline) and the resulting decreased Hb–O_2_ affinity, if arterial *P*_O_2__ is above 80 mmHg, a Bohr shift will result in a minimal decline in Hb saturation of only 3% or less (Fig. 1B).

### Venous Hb saturation and the Bohr effect

Provided arterial saturation is constant, saturation in venous blood is primarily determined by tissue metabolic rate and tissue O_2_ extraction from blood. The greater the tissue O_2_ consumption, the greater the diffusion of CO_2_ back into the tissue capillaries, resulting in a lower blood pH, decreased Hb–O_2_ affinity and enhanced release of O_2_ from Hb. Tissue O_2_ extraction, facilitated by the Bohr shift, will result in a lower venous saturation. The largest differences in calculated changes in saturation between pH 7.5–7.4 and pH 7.5–7.3 occur between 20 and 40 mmHg (Fig. 1B). If *P*_O_2__ is high enough (>40 mmHg) or low enough (<20 mmHg), differences in saturation due to changes in Hb–O_2_ affinity are minimized (Fig. 1B).

### Goals

To explore the effect of changing pH on calculated blood saturation profiles during the dive and during the dynamic recovery phase (the final ascent and the transition from apnea to eupnea) of diving emperor penguins, we first assessed, separately, the effects of increased blood CO_2_ levels and increased blood [lactate] on blood pH in blood samples collected opportunistically in prior field studies. We then determined the most appropriate lower limit for pH during dives and surface intervals of emperor penguins. Second, once we selected the lower limit for pH, we examined the potential effects of this pH on (a) final arterial and venous saturation and the remaining unused blood O_2_ stores at the end of the dive, and (b) the blood O_2_ store depletion rate and its contribution to diving metabolic rate (which had been calculated previously based on an end-of-dive pH of 7.40; Meir and Ponganis, 2009). Third, based on those data and data from prior studies, we calculated total body O_2_ store depletion for dives of 5–6 min (near the ADL) and dives of 10–12 min to determine the quantity of blood O_2_ consumed and also the quantity of O_2_ required to replenish those O_2_ store deficits during the apnea–eupnea transition at the end of a dive. Fourth, in a short series of routine shallow dives, and in the longest dives in which we had *P*_O_2__ data, we examined differences in the Hb saturation profiles calculated at pH 7.5 and at the lower limit pH as upper and lower bounds for saturation throughout the dive and post-dive recovery periods. In addition, we also examined the hypothetical effect of the lower Hb–O_2_ affinity of the giant fulmar on saturation values calculated from the *P*_O_2__ profiles of emperor penguins. These results will help us to elucidate the effect of a lower pH and the role of the Bohr effect and pH on our estimates of arterial saturation and venous saturation during the dive and on the rate of recovery of both arterial and venous saturation to baseline levels in the post-dive period.

## MATERIALS AND METHODS

### Research camp

The data analyzed in this investigation were collected in prior studies (2001–2008); the methods have been previously described in detail (Meir and Ponganis, 2009; Ponganis et al., 2007, 2009). Briefly, non-breeding emperor penguins, *Aptenodytes forsteri* Gray 1844, temporarily captive at an isolated dive hole research camp in McMurdo Sound, Antarctica, were equipped with time depth recorders and either indwelling *P*_O_2__ electrodes or indwelling blood sampling catheters. The birds were allowed to dive freely for 1–2 days during which arterial or venous data were collected with a backpack *P*_O_2__ recorder or with a backpack custom blood sampler. Deployment and removal of devices were conducted under general anesthesia. Birds were released at the end of the study at the McMurdo Sound ice edge. All studies were approved through a UCSD IACUC protocol and Antarctic Treaty Permit.

### Blood sample collection and analysis

Blood samples were collected opportunistically from indwelling arterial or venous catheters during anesthesia, at rest, during dives, and during restraint for blood sampler attachment/removal during isolated dive hole studies in 2004–2008 (Meir and Ponganis, 2009; Ponganis et al., 2007, 2009). *P*_CO_2__, pH, *P*_O_2__ and [lactate] analyses of blood samples were performed with a Series 200 i-STAT portable blood gas analyzer (Abbott Point of Care Inc., East Windsor, NJ, USA).

### Assessment of CO_2_ and lactate effects on pH

To assess the effects of CO_2_ and [lactate] on pH separately, we first identified and evaluated previously collected blood sample data that included pH, *P*_CO_2__, *P*_O_2__ and [lactate]. Next, we divided the blood samples into two groups by a resting [lactate] criterion of 1.5 mmol l^−1^ (Ponganis et al., 1997, 2009). Because blood [lactate] does not increase after dives <ADL or during dives even as long as 10 min (Ponganis et al., 1997, 2009), this group of blood samples with [lactate]<1.5 mmol l^−1^ was relevant to evaluation of a lower bound for blood pH during dives and during surface intervals after dives <5.6 min ADL. In contrast, during surface intervals for dives >ADL, blood [lactate] in unrestrained emperor penguins peaked within 5–12 min after surfacing, ranging from 4 to 13 mmol l^−1^ after dives of 6–12 min duration (Ponganis et al., 1997). Blood [lactate] declined to baseline levels from peak values within about 10 min, approximately 0.6 mmol l^−1^ min^−1^ (Ponganis et al., 1997). Therefore, the group of blood samples with [lactate]>1.5 mmol l^−1^ were most pertinent to evaluation of a lower bound for blood pH during the surface interval, especially after dives >ADL.

We assessed the effect of CO_2_ on pH in the group with <1.5 mmol l^−1^ [lactate] and the effect of lactate on the blood samples with >1.5 mmol l^−1^ [lactate] and <70 mmHg *P*_CO_2__ using mixed effects models in R (v. 4.4.2, http://www.R-project.org/) using packages lme4, lmerTest and MuMIn (https://CRAN.R-project.org/package=MuMIn; http://CRAN.R-project.org/package=lme4; Kuznetsova et al., 2017). Residuals were checked to confirm model requirements. In all models, the response variable was pH and the random variable was penguin ID. Fixed effects included *P*_CO_2__, [lactate], *P*_O_2__, body mass, conditions of sampling (resting, intradive blood sampler and under anesthesia) and sample site (arterial or venous). For the group with [lactate]>1.5 mmol l^−1^, the variable conditions of sampling were not included because there were too few samples for each condition (Table S2). To find the best model, we compared Akaike's information criterion (AIC) values among all models. If there was no significant difference in AIC between models (ΔAIC*<*2), we selected the simplest model (fewest variables). We obtained marginal and conditional *r*^2^ values to determine goodness of fit (Nakagawa and Schielzeth, 2013). To determine the lower pH limit, we examined the lowest pH values from the two groups.

### *P*_O_2__ and saturation profiles

Arterial *P*_O_2__, venous *P*_O_2__ and depth profiles were obtained from studies in 2001–2008 (Meir and Ponganis, 2009; Ponganis et al., 2007, 2009). The O_2_–Hb dissociation curves determined by Meir and Ponganis (2009) were used to convert *P*_O_2__ profiles to Hb saturation profiles. Saturation profiles were constructed using a pH of 7.5 (resting pH), pH 7.4 (used in prior studies) and the lower limit of pH 7.3 determined in this study. To examine the hypothetical effect of a Hb with lower Hb–O_2_ affinity on *P*_O_2__ profiles of emperor penguins, the Hill plot equation of the giant fulmar dissociation curve {log[*S*_O_2__/(100–*S*_O_2__)]=2.74683×log(*P*_O_2__)−4.52219} was developed from the dissociation curve figure of Milsom et al. (1973) (*S*_O_2__=Hb–O_2_ saturation).

### Analyses of saturation and blood O_2_ content

Start-of-dive arterial and venous saturation were calculated at pH 7.5. End-of-dive arterial and venous saturation calculated using pH 7.3 and 7.4 were compared to evaluate the difference in end-of-dive values, the magnitude of blood O_2_ depletion and the contribution to diving metabolic rate (DMR). Continuous arterial and venous saturation profiles were constructed at pH 7.5 and 7.3 to assess the upper and lower bounds of saturation calculated from *P*_O_2__ profiles with use of the respective O_2_–Hb dissociation curves. From these profiles, differences in the magnitude of blood O_2_ depletion, and of blood O_2_ restoration were examined during the apnea-to-eupnea transition (surface period).

As in Meir and Ponganis (2009), the total oxygen content (ml O_2_ dl^−1^) formula [(fraction Hb saturation×1.34×18.3)+(0.003×*P*_O_2__)] assumed 1.34 ml O_2_ g^−1^ Hb, a [Hb] of 18.3 g dl^−1^ and a dissolved O_2_ content (ml O_2_ dl^−1^) of 0.003×*P*_O_2__ (mmHg). Net arterial and venous O_2_ store depletion (ml O_2_ kg^−1^) were based on the difference between initial and final O_2_ content, and a blood volume of 100 ml kg^−1^, with 0.33 blood volume arterial and 0.67 blood volume venous (Meir and Ponganis, 2009). The arterial and venous O_2_ store depletion rates were calculated by dividing net (arterial or venous) O_2_ store depletion by dive duration. The blood O_2_ store contribution to DMR was estimated from the sum of the mean arterial and venous values.

### Calculation of total body O_2_ store contribution to DMR

The total body O_2_ store contribution to DMR was estimated from the calculated blood contributions and from previous estimates of the respiratory and muscle O_2_ store contributions (Williams et al., 2011, 2021). The respiratory O_2_ store contribution was based on representative estimates of the respiratory O_2_ fractions and respiratory air volume (70 ml air kg^−1^) for shallow dives of these durations (Sato et al., 2011; Stockard et al., 2005; Williams et al., 2021). The muscle O_2_ store contribution was evaluated in both the chest (pectoralis–supracoracoideus muscle complex) and leg muscles (Williams et al., 2011). The chest muscle O_2_ store contribution to DMR was based on a myoglobin (Mb) desaturation rate of 14.4% min^−1^ during dives up to 5–6 min in duration (Williams et al., 2011). The mean net Mb desaturation rate in longer dives was 9.8% min^−1^, about 32% slower. Consequently, we assumed a mean chest muscle O_2_ store depletion rate of 3.1 ml O_2_ kg^−1^ min^−1^ during 5–6 min dives, and 2.3 ml O_2_ kg^−1^ min^−1^ in 10–12 min dives, based on Mb concentration, relative muscle mass (i.e. fraction of body mass) and the mean Mb desaturation rates in those dives (Williams et al., 2011). The O_2_ contribution of the leg muscles was based on Mb concentration, relative muscle mass and a resting tissue metabolic rate (Williams et al., 2011).

### Data processing, graphics and statistics software

Calculations of Hb saturation and O_2_ store depletion were conducted in Excel (Microsoft, Redmond, WA, USA). Figure plots of depth, Hb saturation and *P*_O_2__ profiles and of blood pH versus blood *P*_CO_2__ or [lactate] were created with Origin (version 7.0, OriginLab, Northampton, MA, USA). Because of the lack of normality (Shapiro–Wilk test in Origin), non-parametric analyses (Wilcoxon signed-rank test) of differences (pH 7.4 versus 7.3) in final Hb saturation, in net depletion of arterial and venous O_2_ stores and in arterial and venous contributions to metabolic rate were conducted in SPSS (https://www.socscistatistics.com). Arterial and venous saturation recovery times (normal distribution) were assessed with a two-sample Student's *t*-test (Origin). Unless otherwise reported, results are expressed as means±s.e.m. Statistical significance was assumed at *P*<0.05.

## RESULTS

Forty-seven blood samples from 18 penguins were used to determine the lower limit for pH (1) during and at the end of dives and (2) at the surface. Tables S1 and S2 provide collection and analysis information for both groups of samples, including pH, *P*_CO_2__, *P*_O_2__, [lactate], as well as collection year, penguin ID, body mass, sample site (arterial/venous) and comments on the sampling conditions.

**Fig. 2.**
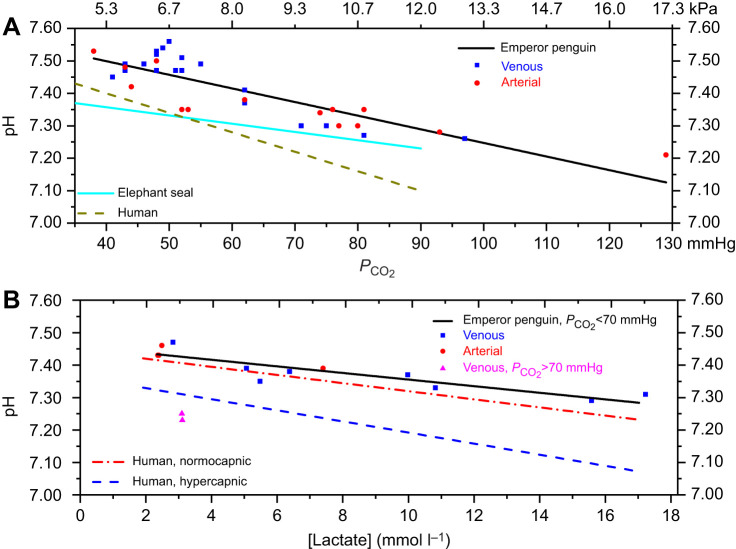
**Blood pH responses to increases in the partial pressure of carbon dioxide (*P*_CO_2__) or in lactate concentration ([lactate]) in emperor penguins.** (A) Blood pH decreased significantly with increased *P*_CO_2__ in emperor penguin blood samples with [lactate]<1.5 mmol l^−1^ (*n*=32, see Results; Table S1). Also included are regression lines for elephant seals (Stockard et al., 2007) and humans (Brackett et al., 1965). (B) Blood pH decreased significantly with increased [lactate] in emperor penguin blood samples with *P*_CO_2__<70 mmHg and [lactate]>2.0 mmol l^−1^ (*n*=12, see Results; Table S2). Also included are regression lines for humans (Kato et al., 2005).

Blood samples with [lactate]<1.5 mmol l^−1^ were used to determine the lower limit for pH during and at the end of dives. The best model for this group included *P*_CO_2__ and sampling conditions. Blood pH was significantly lower with increasing *P*_CO_2__ and in samples taken during dives or under anesthesia, with individual providing a small effect (marginal *r*^2^, *r*^2^_m_=0.86; conditional *r*^2^, *r*^2^_c_=0.95) (Fig. 2A). For every 10 mmHg increase in *P*_CO_2__, pH decreased by 0.034 units. In 12 samples taken during rest, pH was 7.51±0.03. In all but five of the 32 samples, pH was ≥7.30. In four of those five samples, *P*_CO_2__ was ≥93 mmHg, an unlikely value in an awake or diving animal. Based on these data (Fig. 2A; Table S1), and the lack of lactate elevation during a dive (Ponganis et al., 2009), a pH of 7.3 was chosen as a lower bound with which to calculate end-of-dive saturation and blood O_2_ depletion during dives.

The lower limit for pH during surface intervals was determined from samples with [lactate]>1.5 mmol l^−1^. The best model for this group included [lactate] as the only fixed effect. As [lactate] increased, pH decreased significantly (*r*^2^_m_=0.795, *r*^2^_c_=0.92), with pH decreasing 0.09 units for every 10 mmol l^−1^ lactate increase (Fig. 2B). In 11 blood samples with a *P*_CO_2__ less than 70 mmHg, mean pH was 7.29±0.04. In three samples with a [lactate] above 10 mmol l^−1^, pH dropped to a mean of 7.31±0.02.

For samples with [lactate]>1.5 mmol l^−1^, pH was ≥7.29 except for two anesthetized animals (7.23, 7.25) with *P*_CO_2__ values of 72 and 85 mmHg, respectively. Consequently, on the basis of these data (Fig. 2B; Table S2), a pH of 7.3 was chosen as a lower bound with which to calculate Hb saturation and blood O_2_ repletion during surface intervals.

### Comparison of end-of-dive saturation using pH 7.3 versus 7.4

**Fig. 3.**
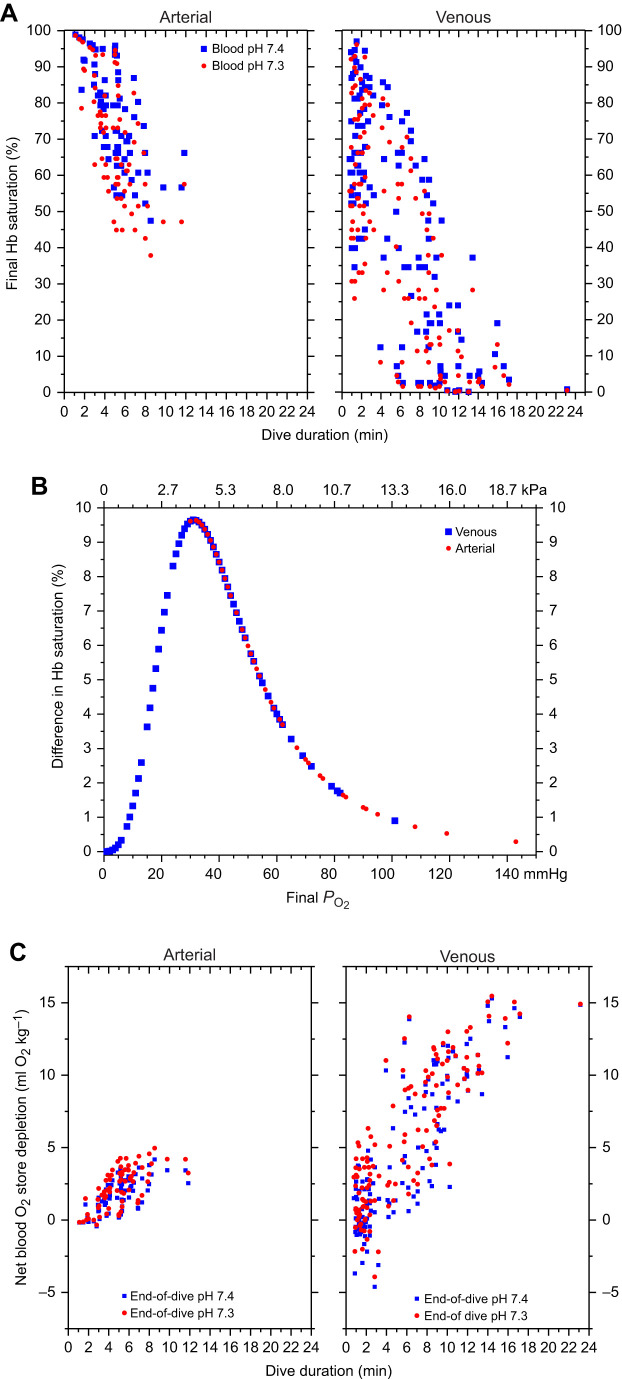
**Effects of pH 7.4 versus 7.3 in calculations of O_2_ transport parameters.** (A) Final arterial and venous Hb saturation calculated with pH 7.4 and 7.3 O_2_–Hb dissociation curves versus dive duration. (B) Differences in final arterial and venous Hb saturation versus end-of-dive *P*_O_2__. (C) Net arterial and venous O_2_ store depletion (ml O_2_ kg^−1^) at pH 7.4 and 7.3 versus dive duration.

Saturation during dives was calculated from 71 arterial *P*_O_2__ profiles and 130 venous *P*_O_2__ profiles. End-of-dive arterial and venous saturation values at pH 7.3 versus 7.4 were significantly different (Wilcoxon signed-rank test; arterial *z*=−7.32; venous *z*=−9.78). The pH 7.3 saturation values were up to 10% lower dependent on the end-of-dive *P*_O_2__ (Fig. 3; Fig. S1). The median percentages of the initial O_2_ store remaining at the end of the dive were 79.0% and 70.2% for the arterial store at pH 7.4 and 7.3, respectively, and 73.9% and 60.9% for the venous store at pH 7.4 and 7.3, respectively (Table 1). The resulting differences in the net depletion (ml O_2_ kg^−1^) of the arterial and venous O_2_ stores were significantly different (Wilcoxon-signed rank test; arterial *z*=−7.32, venous *z*=−9.89). Blood O_2_ store depletion rates (ml O_2_ kg^−1^ min^−1^) at pH 7.4 and 7.3 were also significantly different (Wilcoxon-signed rank test; arterial *z*=−7.32, venous *z*=−9.89). The pH 7.3 data for all dives resulted in a 27% increase in calculated blood O_2_ store depletion rate (1.4 versus 1.1 ml O_2_ kg^−1^ min^−1^) (combined arterial and venous medians in Table 1), but only a 5% increase in the total body O_2_ store depletion rate (6.5 versus 6.2 ml O_2_ kg^−1^ min^−1^) estimated with pH 7.4 blood data and the previously calculated respiratory (1.5 ml O_2_ kg^−1^ min^−1^) and muscle (3.6 ml O_2_ kg^−1^ min^−1^) data (Williams et al., 2011, 2021).

**Table 1.** Differences in the magnitude, change and contribution to diving metabolic rate of final blood O_2_ stores based on blood oxygen partial pressure (*P*_O_2__) profile and hemoglobin (Hb) saturation calculated at blood pH 7.4 versus 7.3 in emperor penguins

	Initial (ml O_2_ kg^−1^)	Final (ml O_2_ kg^−1^)		Net depletion (ml O_2_ kg^−1^)		% Net Depletion		Contribution to DMR (ml O_2_ kg^−1^ min^−1^)	
		pH 7.4	pH 7.3	pH 7.4	pH 7.3	pH 7.4	pH 7.3	pH 7.4	pH 7.3
Arterial O_2_ store									
Median	7.9	6.0	5.4	1.7	2.2	21.0	29.8	0.4	0.5
Max.	8.2	8.1	8.1	4.2	5.0	51.9	61.6	0.7	0.9
Min.	7.0	3.9	3.1	−0.4	−0.3	5.9	−4.5	−0.2	−0.1
25% quartile	7.9	5.4	4.7	0.8	1.2	10.7	15.2	0.2	0.2
75% quartile	8.0	7.0	6.7	2.5	3.2	32.0	40.8	0.5	0.6
Venous O_2_ store									
Median	12.8	8.6	7.1	3.6	5.1	26.1	39.1	0.7	0.9
Max.	15.8	16.1	16.0	15.3	15.5	100.0	100.0	3.2	4.5
Min.	6.4	0.0	0.0	−4.6	−3.9	−57.9	−40.2	−4.2	−2.5
25% quartile	10.8	2.9	2.0	0.6	1.8	4.3	16.4	0.2	0.5
75% quartile	14.4	12.1	10.9	9.6	10.1	78.9	85.4	1.1	1.3

Arterial and venous O_2_ stores were calculated for dives of 1–23.1 min duration (Meir and Ponganis, 2009; Ponganis et al., 2007, 2009). Initial arterial and venous O_2_ stores were estimated with Hb saturation calculated at pH 7.5. The net depletion of arterial and venous stores was calculated as the difference between initial and final stores. The percentage net depletion was calculated as the difference between the initial and final O_2_ stores divided by the initial store. The arterial and venous contributions to diving metabolic rate (DMR) were calculated by dividing net depletion during each dive by the dive duration. Differences between pH 7.4 and 7.3 for final stores, net depletion and contribution to DMR were significant (Wilcoxon signed-rank test, *P*<0.05). *N*=71 dives arterial, *N*=130 dives venous. O_2_ store calculations and assumptions are in the Materials and Methods. Max., maximum; Min., minimum.

In dives of 5–6 min duration (Table S3), mean end-of-dive arterial saturation and the remaining arterial O_2_ store were both approximately 7% lower for calculations at pH 7.3 versus pH 7.4. Mean end-of-dive venous saturation and the remaining venous O_2_ store were 6% and 8.5% less at pH 7.3. The mean total blood O_2_ store contributions to DMR were 1.6 and 1.8 ml O_2_ kg^−1^ min^−1^ for calculations at pH 7.4 and 7.3, respectively.

In dives of 10–12 min duration, Hb saturation (calculated only at pH 7.3) reached mean end-of-dive arterial and venous values of 51% and 9%, respectively (Table S4). These values resulted in unused end-of-dive arterial and venous O_2_ stores that were 52% and 13% of the start-of-dive values. In comparison, in 5–6 min dives, the mean unused arterial and venous O_2_ stores at the end of the dive (calculated at the same pH – 7.3) were 69% and 39%, respectively. The blood O_2_ store contribution to DMR for 10–12 min dives was 1.4 ml O_2_ kg^−1^ min^−1^, 22% less than that calculated at pH 7.3 for 5–6 min dives, and 13% less than that calculated at pH 7.4 for 5–6 min dives.

### Comparison of saturation profiles using pH 7.3 versus 7.5 dissociation curves

**Fig. 4.**
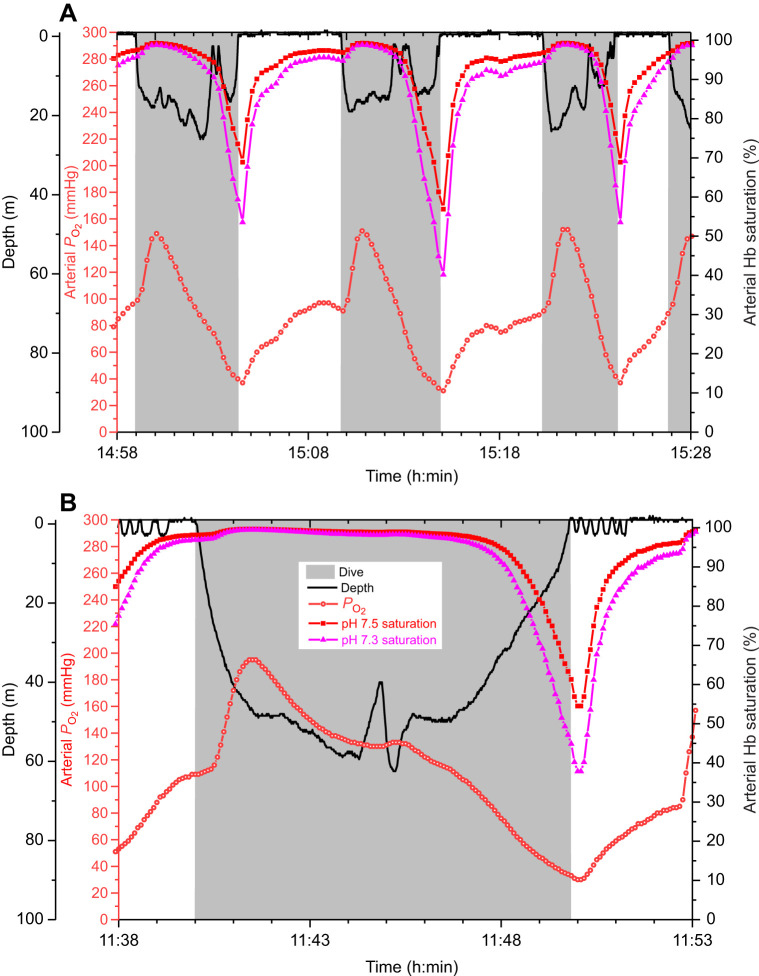
**Depth profiles, arterial *P*_O_2__ profiles and arterial Hb saturation profiles at pH 7.5 and 7.3 in emperor penguins.** (A) Data for three consecutive dives of 4–7 min duration (EP9-2007). (B) Data for a 9.8 min dive (EP1–2008).

**Fig. 5.**
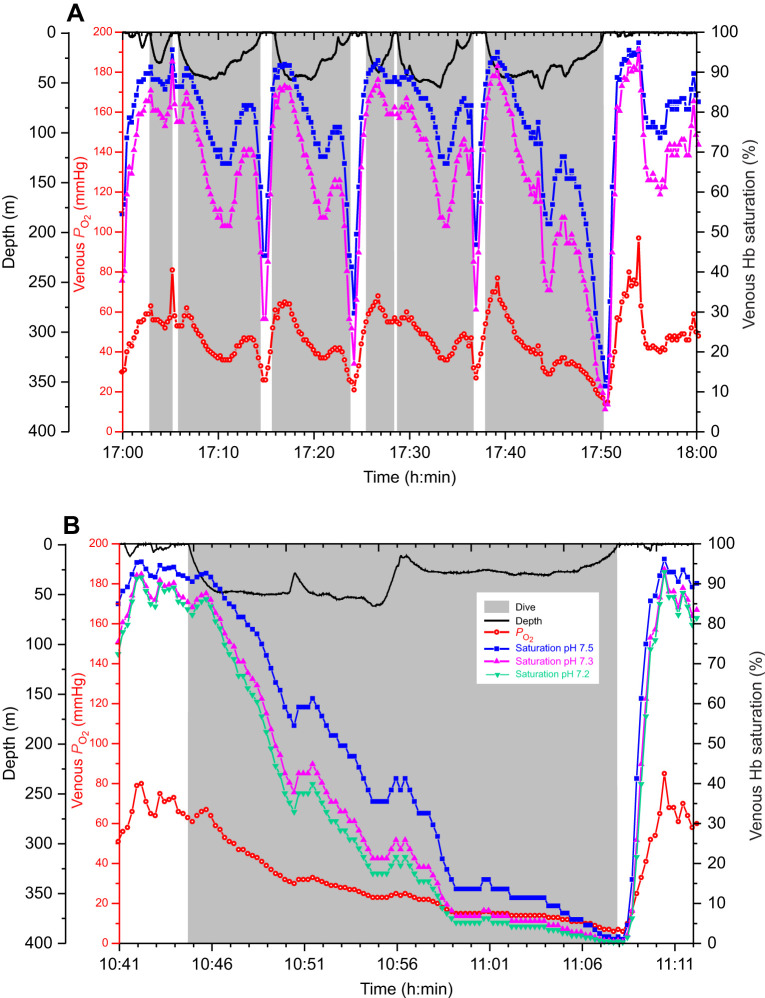
**Depth profiles, venous *P*_O_2__ profiles and venous Hb saturation profiles calculated at pH 7.5, 7.3 and 7.2 in an emperor penguin.** (A) Data for a series of 2–10 min dives at pH 7.5 and 7.3 (EP19-2007). (B) Venous Hb saturation profiles calculated at pH 7.5, 7.3 and 7.2 throughout a 23.1 min dive (EP19-2007).

Arterial and venous saturation profiles constructed at pH 7.5 and 7.3 demonstrated differences in the upper and lower bounds of saturation (Figs 4 and 5). Arterial saturation at pH 7.3 was well maintained (>90%) during most of the dive, even in dives as long as 9.8 min (Fig. 4B). Although end-of-dive saturation was lower than in the pH 7.5 profile, saturation in both profiles only began to decline during final ascent. Venous saturation profiles at pH 7.5 and 7.3 in dives of 2.3–12.3 min duration demonstrated larger differences in saturation throughout the dive than in arterial saturation profiles (Fig. 5A). The magnitude of these differences was dependent on the blood *P*_O_2__ value as demonstrated for end-of-dive values (Fig. 3B).

At the surface, restoration of blood O_2_ to baseline levels of 90% and 80% for arterial and venous saturation, respectively, occurred within 3 min regardless of pH. The mean time for arterial saturation recovery at pH 7.3 (16 dives in two birds) was 1.99±0.46 min and that for venous saturation recovery at pH 7.3 (13 dives in one bird) was 1.60±0.13 min. These mean recovery times were 69% and 60% longer than those at pH 7.5 (1.20±0.05 min and 1.10±0.10 min) for arterial Hb saturation and venous saturation, respectively. Recovery times at pH 7.5 and pH 7.3 were significantly different for both arterial and venous data (two-sample Student’s *t*-test, *P*<0.01).

In one of the longest dives reported for an emperor penguin (23.1 min, Fig. 5B), venous saturation profiles at pH 7.5, 7.3 and 7.2 demonstrated that saturation values were quite close regardless of pH during the final 3 min of the dive when venous *P*_O_2__ was <10 mmHg. In addition, recovery of saturation during the surface period was rapid regardless of pH. After the end of this extreme dive, 80% venous saturation was reached within 1.9 min for pH 7.5 and 2.2 min for both pH 7.3 and 7.2.

In saturation profiles generated with use of the giant fulmar O_2_–Hb dissociation curve from these *P*_O_2__ profiles of emperor penguins, 56% (9 of 16 dives) of arterial profiles and 77% (10 of 13 dives) of venous profiles did not reach the respective 90% and 80% saturation baseline prior to the next dive. In those that did reach the baseline, arterial recovery times were 47–432% longer than those for the pH 7.3 emperor penguin O_2_–Hb dissociation curve, while venous times were 12–72% longer.

## DISCUSSION

### Upper and lower bounds of blood pH during and after dives

#### Effect of *P*_CO_2__ on pH and determination of upper and lower blood pH values during dives

In blood samples with blood [lactate]<1.5 mmol l^−1^ (Table S1), the highest pH values were from samples collected when penguins were resting. Based on the average of those values at rest, we considered a pH of 7.5 as a reasonable upper bound for pH during dives. To evaluate a lower bound for blood pH during dives, we examined the relationship between pH and *P*_CO_2_ _(Fig. 2A) because *P*_CO_2__, but not [lactate], increases during dives. The significant decrease in pH in response to increased *P*_CO_2__ was similar to that in several marine mammals, but greater than that in elephant seals (*Mirounga angustirostris*) and less than that in humans (Brackett et al., 1965; Noren et al., 2012; Ponganis et al., 2017; Stockard et al., 2007). A pH of <7.30 during routine dives of 5–10 min is unlikely for three reasons: (1) pH was >7.28 even in samples with a *P*_CO_2__ near 80 mmHg (Fig. 2A), (2) intradive blood [lactate] remained below or near resting levels (<2.0 mmol l^−1^) as far as 10 min into a dive (Ponganis et al., 2009), and (3) intradive pH was above 7.35 with *P*_CO_2__ values between 48 and 62 mmHg (Ponganis et al., 2009). Therefore, we think that application of the pH 7.5 and 7.3 dissociation curves to a *P*_O_2__ profile provides reasonable upper and lower bounds for saturation during routine dives.

#### Effect of [lactate] on pH and determination of upper and lower blood pH bounds during surface intervals

For samples with blood [lactate]>1.5 mmol l^−1^ (Table S2), pH decreased significantly as blood [lactate] increased (Fig. 2B). The pH decrease of 0.09 pH units per 10 mmol l^−1^ increase in [lactate] was similar to that in exercising humans (Kato et al., 2005). These samples were collected opportunistically primarily when penguins were restrained before or after dives. Because blood [lactate] can increase secondary to handling and restraint (Le Maho et al., 1992), the samples obtained during restraint were not representative of true ‘post-dive’ [lactate], but they did help to provide a larger range of blood values to evaluate the pH–[lactate] relationship (Table S2).

Blood pH was rarely below 7.30 despite elevations in blood [lactate] as high as 17 mmol l^−1^ (Fig. 2B). Only three of 14 samples had a pH <7.30 and two of these were associated with a *P*_CO_2_ _>70 mmHg (Table S2). The two venous samples with the highest [lactate] were obtained during restraint while a backpack blood sampler was removed after long dives (Table S2). Despite [lactate] of 15.4 and 17.0 mmol l^−1^, and with *P*_CO_2__ of 37 and 38 mmHg, blood pH values were 7.29 and 7.31, respectively. This 0.2 pH unit change from baseline was slightly less than the 0.27 pH unit change in a Weddell seal (*Leptonychotes weddellii*) that had a similar blood [lactate] of 15 mmol l^−1^ but higher *P*_CO_2__, near 50 mmHg (Qvist et al., 1986).

The low *P*_CO_2__ values of 37–38 mmHg in the two samples with the highest lactate values were obtained >9 min after long dives (Table S2), suggesting that washout and removal of CO_2_ is relatively rapid, and is accomplished within 10 min even after dives of 10.7–17.3 min. In contrast, lactate washout in unrestrained penguins post-dive (see Materials and Methods) appeared slower than CO_2_ washout with peak [lactate] values 5–10 min post-dive, and a return to baseline about 10 min later (Ponganis et al., 1997). Given these findings, we consider the pH 7.5 and 7.3 Hb saturation profiles as appropriate upper and lower bounds during the surface interval.

### The effect of different pH levels on calculated Hb saturation profiles

Based on these results, we reconstructed saturation profiles at pH 7.5, 7.4 and 7.3 from blood *P*_O_2__ profiles previously collected from spontaneous foraging dives of emperor penguins diving at an isolated dive hole (Meir and Ponganis, 2009; Ponganis et al., 2009, 2007). These calculated saturation profiles represent our estimated upper and lower limits of saturation for the *P*_O_2__ values recorded during the dive. We remind readers that the changes in blood pH, *P*_O_2__ and saturation during a dive are due to the interplay of gas exchange in the lungs, tissue O_2_ delivery by the circulatory system, and tissue metabolic rate (Meir et al., 2008; Ponganis et al., 2025; Williams et al., 2011). We are simply using pH to estimate saturations from *P*_O_2__ profiles that are the result of those processes.

### How pH affects saturation profiles calculated from *P*_O_2__ profiles

#### End-of-dive saturation values

Compared with a blood pH of 7.4, a pH of 7.3 had significantly lower end-of-dive saturation values and higher blood O_2_ depletion rates for dives of all durations in the study (Table 1). The net changes in the combined arterial and venous O_2_ stores calculated with a final pH of 7.3 versus 7.4 resulted in a 27% increase in the median calculated blood O_2_ store depletion rate at pH 7.3 and a 5% increase in the total body O_2_ store depletion rate (6.5 versus 6.2 ml O_2_ kg^−1^ min^−1^). With a final pH of 7.3, the blood O_2_ store was not depleted at the 5.6 min ADL or for dives as long as 12 min (Table 1, Fig. 3). The total body O_2_ store depletion rate calculated at pH 7.3 (6.5 ml O_2_ kg^−1^ min^−1^) was still relatively low, equivalent to resting metabolic rates predicted by allometric equations and also measured in emperor penguins at rest in water flume (Kooyman and Ponganis, 1994).

#### Saturation values for 5–6 min dives

Blood O_2_ store calculations for 5–6 min dives (near the 5.6 min ADL) resulted in a combined arterial and venous O_2_ store contribution to DMR of 1.6 and 1.8 ml O_2_ kg^−1^ min^−1^, respectively, for a final pH of 7.4 and 7.3 (Table S3). Although greater than values calculated for all dives in the study, blood O_2_ stores at the end of 5–6 min dives were far from depleted at either pH (Table S3). We consider these blood O_2_ store depletion rates (1.6 and 1.8 ml O_2_ kg^−1^ min^−1^) more realistic for dive durations near the ADL than our estimates of 1.1 and 1.4 ml O_2_ kg^−1^ min^−1^ at each pH for all dives (dive duration: 1–23 min) (Table 1). Our current estimates of the blood O_2_ store contribution to DMR for 6 min dives were 145% and 129% (pH 7.4 and 7.3) of the previous values for all dives.

#### Saturation values for 10–12 min dives

Given the longer durations of 10–12 min dives (approximately twice the ADL), blood O_2_ calculations were based only on an end-of-dive pH of 7.3 (Table S4). Blood O_2_ stores were still not depleted during these dives. The blood O_2_ store contribution to DMR during a 10 min dive was 1.4 ml O_2_ kg^−1^ min^−1^, equivalent to that previously calculated at pH 7.3 with data for all dives (Table 1). We consider this value more realistic for a 10 min dive because it is based on data from dives of this duration. This blood O_2_ contribution to DMR for a 10 min dive was 0.4 ml O_2_ kg^−1^ min^−1^ less than that calculated with the same final pH (7.3) for a 6 min dive.

### Total body O_2_ store utilization and depletion

These blood O_2_ store calculations in dives of specific duration in conjunction with prior studies of respiratory and muscle O_2_ stores in diving emperor penguins provided the basis for an overview of total body O_2_ store utilization and depletion during dives near the 5.6 min ADL and dives of almost 2-fold greater duration (Table 2). Importantly, as described in Materials and Methods, both respiratory and muscle O_2_ depletion rates decrease as dive duration increases (Sato et al., 2011; Stockard et al., 2005; Williams et al., 2011, 2021).

**Table 2.** Net O_2_ store contribution to DMR and depletion of O_2_ stores in dives of 5–6 and 10–12 min duration in emperor penguins

	5–6 min dives		6 min dive	10–12 min dives		10 min dive
	Initial O_2_ store (ml O_2_ kg^−1^)	Net O_2_ store contribution to DMR (ml O_2_ kg^−1^ min^−1^)	O_2_ store consumed (ml O_2_ kg^−1^)	Initial O_2_ store (ml O_2_ kg^−1^)	Net O_2_ store contribution to DMR (ml O_2_ kg^−1^ min^−1^)	O_2_ store consumed (ml O_2_ kg^−1^)
Arterial	7.3	0.4	2.4	8.0	0.4	4.0
Venous	13.1	1.4	8.4	11.9	1.0	10.0
Respiratory	12.2	1.6	9.6	12.2	0.9	9.0
Muscle–chest	21.4	3.1	18.6	21.4	2.0	20.0
Muscle–legs	3.0	0.5	3.0	3.0	0.3	3.0
Total	57.0	7.0	42.0	56.5	4.6	46.0

The net total body O_2_ store contribution to DMR of 5–6 min dives and 10–12 min dives was 6% more and 30% less, respectively, than the average 6.5–6.6 ml O_2_ kg^−1^ body mass min^−1^ value calculated with the data of all dives from this and prior studies (Williams et al., 2011, 2021). The net total body O_2_ store contribution to DMR of 5–6 min dives was 52% greater than that of 10–12 min dives. Initial total body O_2_ stores were 57 ml O_2_ kg^−1^, based on the blood data in this paper (with final pH 7.3 for both sets of dive durations) and previous data and calculations for respiratory and muscle O_2_ stores (Williams et al., 2011, 2021). Total body O_2_ stores were not depleted even in dives of almost twice the 5.6 min aerobic dive limit (dive duration associated with the onset of post-dive lactate accumulation; Ponganis et al., 1997): 44% of the initial total body O_2_ stores remained after dives of 6 min duration; 19% remained after dives of 10 min duration; 47% and 30% of the blood O_2_ store remained after 6 and 10 min dives, respectively. During the apnea-to-eupnea transition after a dive, restoration of total body O_2_ stores required only an additional 4 ml O_2_ kg^−1^ body mass after a 10 min dive versus a 6 min dive. Arterial and venous O_2_ store depletion rates were calculated as in Materials and Methods and Tables S3 and S4 (with end-of-dive pH 7.3). Respiratory and muscle O_2_ store depletion rates were based on the assumptions, calculations and figures for dives of these durations in Williams et al. (2011, 2021). The O_2_ store consumed was the product of the net O_2_ store contribution to DMR×dive duration. Although the muscle O_2_ store was not completely consumed in the calculation, final myoglobin saturation was less than the 20% threshold for the onset of muscle lactate accumulation (Scholander, 1940; Scholander et al., 1942).

#### O_2_ utilization for 5–6 min dives

During these 5–6 min dives, the O_2_ contribution of the chest muscle (pectoralis–supracoracoideus muscle complex) to DMR was greatest among the O_2_ stores (Table 2). O_2_ use by the chest and leg muscles comprised over 50% of the total O_2_ consumption. The contributions of the venous and respiratory O_2_ stores to DMR were almost equivalent to each other. The total contribution of all the O_2_ stores to DMR in a 6 min dive was 7.0 ml O_2_ kg^−1^ min^−1^, 113% of that previously calculated with mean values of all dives (Williams et al., 2021). At the end of 5–6 min dives, 25% of the initial total body O_2_ store (57 ml O_2_ kg^−1^; Table 2) remained. (Note that the respiratory O_2_ store for these shallow dives is less than that often calculated for deep dives because of the difference in start-of-dive respiratory air volumes; Sato et al., 2011.) In addition, blood O_2_ store depletion rates in Table 2 were based on an end-of-dive pH of 7.3; Table S3). The O_2_ store deficit required to restore all body O_2_ stores to pre-dive levels was 42 ml O_2_ kg^−1^.

#### O_2_ utilization for 10–12 min dives

Although twice as long as the 5–6 min dives, the 10–12 min dives consumed only 9.5% more O_2_ (Table 2). The largest O_2_ contribution to DMR was from the chest muscle; the chest and leg muscle combined yielded 50% of the O_2_ contribution to DMR. The conservation of O_2_ during these longer dives is attributed to a lower aerobic metabolic rate consistent with (a) a more intense dive response (slower heart rate and decreased tissue perfusion) in longer dives (Meir et al., 2008), (b) anaerobic metabolism in longer dives (Williams et al., 2012), and (c) decreased or more efficient stroke effort (Williams et al., 2012). The total body O_2_ store contribution to DMR during a 10 min dive was 4.6 ml O_2_ kg^−1^ min^−1^, 74% of that calculated previously with mean values of all dives (Williams et al., 2021), and 66% of that calculated above for 6 min dives. The O_2_ store deficit required to restore all body O_2_ stores to pre-dive levels after a 10 min dive was 46 ml O_2_ kg^−1^, 4 ml O_2_ kg^−1^ more than after a 6 min dive.

#### Optimal Hb function required for efficient diving

These calculations of the utilization of the different components of the total body O_2_ store demonstrate the importance of blood O_2_ transport and Hb function both during the dive and during the surface period. The progressive depletion of the respiratory and blood O_2_ stores during dives emphasizes the need for optimal Hb function in the transfer of O_2_ from lungs to tissues during the dive, especially during late ascent. Similarly, during the apnea-to-eupnea transition of the surface period, optimal Hb function is required for efficient lung-to-tissue O_2_ transfer, especially for replenishment of the muscle O_2_ store. To restore depleted body O_2_ stores (respiratory, blood and muscle) during the early surface period, 42–46 ml O_2_ kg^−1^ are required for 6 min and 10 min dives, respectively (Table 2). Of this total, about 32–37 ml O_2_ kg^−1^ (for 6–10 min dives, Table 2) needs to be transferred by Hb from the lungs to the blood and muscle O_2_ stores during the surface interval, a time of initial hypoxia followed by tachycardia, hyperventilation and shifting blood pH secondary to washout of CO_2_ and any lactate from muscle and other tissues.

### Higher O_2_-affinity Hb: before and during final ascent to the surface

Regardless of pH, arterial *P*_O_2__ profiles during routine shallow dives of 5–10 min were above 90% during most of the dive (Fig. 4) (Meir and Ponganis, 2009). The position of a high *P*_O_2__ value on the upper end of the O_2_–Hb dissociation curve ensured adequate arterial oxygenation during most of the dive even if the curve shifted as a result of a buildup of CO_2_ and decrease in pH during the dive and final ascent (Butler and Taylor, 1983; Hudson and Jones, 1986; Ponganis et al., 2009). In addition, any potential change in arterial pH was minimized by a decreased release of CO_2_ and lactate into the blood due to the dive response and decreased tissue perfusion during the dive.

We also note that if an emperor penguin had reached a baseline *P*_CO_2__ of 40 mmHg after a dive, but resumed diving with an elevated blood [lactate], arterial oxygenation should be adequate, especially for short duration dives to 20–50 m depth [3–6 atmospheres absolute (ATA)]. First, respiratory *P*_O_2__ will increase with depth and CO_2_ accumulation will be limited by a short dive duration. Second, in blood samples from penguins with [lactate] of 5–10 mmol l^−1^ and *P*_CO_2__ of 43–62 mmHg, pH ranged from 7.39 to 7.35 (Table S1; Fig. 2), well above the pH 7.3 value used to construct the lower bound arterial Hb saturation profile in Fig. 4. Third, it is not uncommon for dives of less than 2 min and less than 50 m in depth to occur during inter-deep-dive intervals of emperor penguins at sea (Kooyman et al., 2020; Sato et al., 2011). Therefore, we conclude that during such short, shallow dives, shifts in the O_2_–Hb dissociation curve due to pH should not impair arterial oxygenation, dive performance or even metabolic processing of lactate.

During the dives in this study, the advantage of a higher-affinity Hb (pulmonary O_2_ extraction and increased O_2_ content at a low *P*_O_2__) occurred primarily during final ascent and during the apnea-to-eupnea transition in early recovery (Figs 4 and 5). On ascent, lung *P*_O_2__ decreases quickly secondary to a low respiratory O_2_ fraction and the decline in ambient pressure. After surfacing, despite washout of CO_2_ and lactate from tissues into venous blood, arterial *P*_O_2_ _climbs steadily in response to tachycardia, hyperventilation, the increase in the respiratory O_2_ fraction and exhalation of CO_2_ (Fig. 4). Although washout of CO_2_ and any lactate from tissues into venous blood during this period decreases pH, enhancing the Bohr effect and tissue O_2_ delivery, pulmonary gas exchange augments the rise in *P*_O_2__ and enhances the removal of CO_2_, minimizing any respiratory acidosis and decrease in the O_2_ affinity of Hb in the pulmonary capillaries.

Although blood O_2_ extraction by tissue (tissue perfusion and tissue O_2_ demand) primarily determines venous saturation, the lower mid-dive values calculated at pH 7.3 versus 7.5 between the two saturation profiles in Fig. 5 would be consistent with a Bohr effect that would promote O_2_ delivery at the lower pH. However, in one of the longest dives reported in emperor penguins (23.1 min; Fig. 5B), it is notable that differences in venous saturation at extremely low *P*_O_2__ during the last few minutes of the dive were minimal regardless of the Hb saturation profile used. As discussed previously, this was consistent with the shape of the O_2_–Hb dissociation curve (Fig. 1).

### Rapid restoration of blood O_2_ during the apnea-to-eupnea transition

During the surface period, the rapid recovery of arterial and venous Hb saturation at pH 7.3 (∼2 min and 1.6 min, respectively) allowed for short surface intervals despite being significantly different (69% and 60% longer, respectively) than those at pH 7.5 (Figs 4 and 5). Even in the longest dive of this dataset (23.6 min), venous Hb saturation recovered to 80% within 2 min (Fig. 5B). If pH decreased to 7.2 because of high blood [lactate] and *P*_CO_2_ _after this extreme dive, venous Hb saturation would still recover within 2 min after surfacing (Fig. 5B).

In addition to hyperventilation and increased cardiac output, optimal Hb function and O_2_ transport underlie these swift increases in arterial and venous saturation and the quick replenishment of the Mb O_2_ store (Williams et al., 2011). Given O_2_ store deficits of 42 and 46 ml O_2_ kg^−1^ after 6 and 10 min dives, respectively, replenishment of respiratory, blood and muscle O_2_ stores within 2 min would require 21–23 ml O_2_ kg^−1^ min^−1^ uptake into the body. An additional 5–7 ml O_2_ kg^−1^ min^−1^ after 6 and 10 min dives, respectively (Table S5), would be required for restoration of the estimated phosphocreatine store depletion in the chest locomotory muscle within 2 min (Williams et al., 2012). The resulting 26–30 ml O_2_ kg^−1^ min^−1^ uptake rate of O_2_ into the body plus increased O_2_ uptake associated with cardiorespiratory costs and metabolic demands of re-perfused organs would probably approach maximum O_2_ consumption rates (44–52 ml O_2_ kg^−1^ min^−1^) of swimming emperor penguins (Kooyman and Ponganis, 1994). Post-dive heart rates similar to or greater than those at maximal swim performance support this suggestion of near-maximal rates of O_2_ uptake during the apnea-to-eupnea transition and again emphasize the value of optimal Hb function during the early surface period (Kooyman and Ponganis, 1994; Meir et al., 2008). Prolongation of surface intervals after longer, and especially extreme dives is probably more dependent on clearance of CO_2_ and lactate as well as replenishment of tissue glycogen stores than on restoration of O_2_ stores (Williams et al., 2012).

**Fig. 6.**
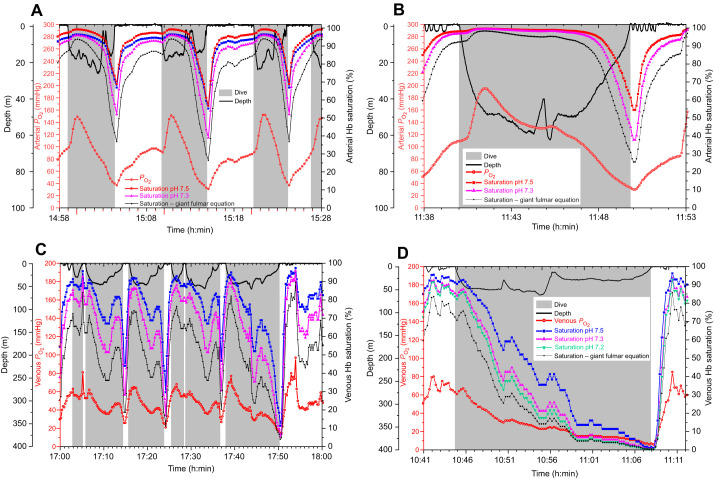
**Comparison of the lower affinity Hb of the giant fulmar with that of the emperor penguin on calculated arterial and venous Hb saturation during dives.** (A,B) Arterial profiles for data in Fig. 4A and B, respectively. (C,D) Venous profiles for data in Fig. 5A and B, respectively.

Lastly, the value of a higher Hb–O_2_ affinity in restoration of the blood O_2_ store is also evident in a hypothetical model in which the *P*_O_2__ profiles in Figs 4 and 5 from dives of emperor penguins were converted to saturation with use of the dissociation curve of the giant fulmar (Fig. 6). Arterial and venous saturation values calculated with the giant fulmar dissociation curve were lower than even the pH 7.3 emperor penguin saturation values throughout the dive cycle (Fig. 6). Not only were start-of-dive saturation and therefore the available blood O_2_ store lower in calculations based on the giant fulmar Hb but also the arterial and venous post-dive recovery to baseline saturation was slower. In over half of these surface intervals, baseline saturation was not even reached prior to the next dive. Despite the limitations of this simple model, these findings support the concept that a critical role of the high Hb–O_2_ affinity and large Bohr effect in the penguin is to enhance blood O_2_ extraction in the lung, contributing to a more rapid increase in restoration of the blood O_2_ store. In general, for transfer of any given quantity of CO_2_ from the pulmonary capillary to the lung, a larger Bohr effect will result in a greater increase in Hb–O_2_ affinity. And, in the periphery, rapid replenishment of the muscle O_2_ store is enhanced by the decreased Hb–O_2_ affinity associated with the decreased blood pH secondary to the washout of CO_2_ and occasionally lactate into muscle capillaries. In short, the apnea-to-eupnea transition is a textbook example of Hb–O_2_ affinity and the Bohr effect.

### Conclusions

We found that a pH of 7.3 was the best available estimate of a lower bound for blood pH during diving of emperor penguins. Based on saturation values calculated from *P*_O_2__ profiles at pH 7.5–7.3, we concluded that (a) arterial oxygenation remained high regardless of pH until late ascent, (b) the median blood O_2_ store depletion rate of all dives increased by 27% with an end-of-dive pH of 7.3 versus 7.4, resulting in a 5% increase in the total body O_2_ store depletion rate, but the blood O_2_ store was not depleted even at dive durations 2 times the ADL, and (c) blood pH and the high affinity and Bohr effect of emperor penguin Hb were especially critical during late ascent and the early surface period. As originally suggested over 50 years ago (Milsom et al., 1973), we postulate that the high Hb–O_2_ affinity and the Bohr effect of emperor penguin Hb provide a higher saturation and enhanced pulmonary O_2_ extraction at low *P*_O_2__ during late ascent, and also enhance pulmonary O_2_ extraction and tissue O_2_ delivery during the flux of CO_2_ from tissues to blood and into the lungs during the early surface period. Tachycardia, hyperventilation and optimal Hb function during the surface period restore arterial, venous and muscle O_2_ stores to baseline within 2 min (Meir and Ponganis, 2009; Stockard et al., 2005; Williams et al., 2011, 2021). The apnea-to-eupnea transition represents the most dynamic period not only of cardiorespiratory function but also of blood O_2_ transport during the dive cycle.

## Supplementary Material

10.1242/jexbio.251044_sup1 Supplementary information
