# Supplementary material for: Blood pH, hemoglobin function and oxygen transport throughout the dive cycle of emperor penguins
Source: J Exp Biol. 2025 Nov 19;228(22):jeb251044. doi: 10.1242/jeb.251044 (PMC12669838; doi:10.1242/jeb.251044)
Supplement: Supplementary information [file jexbio-228-251044-s1.pdf]

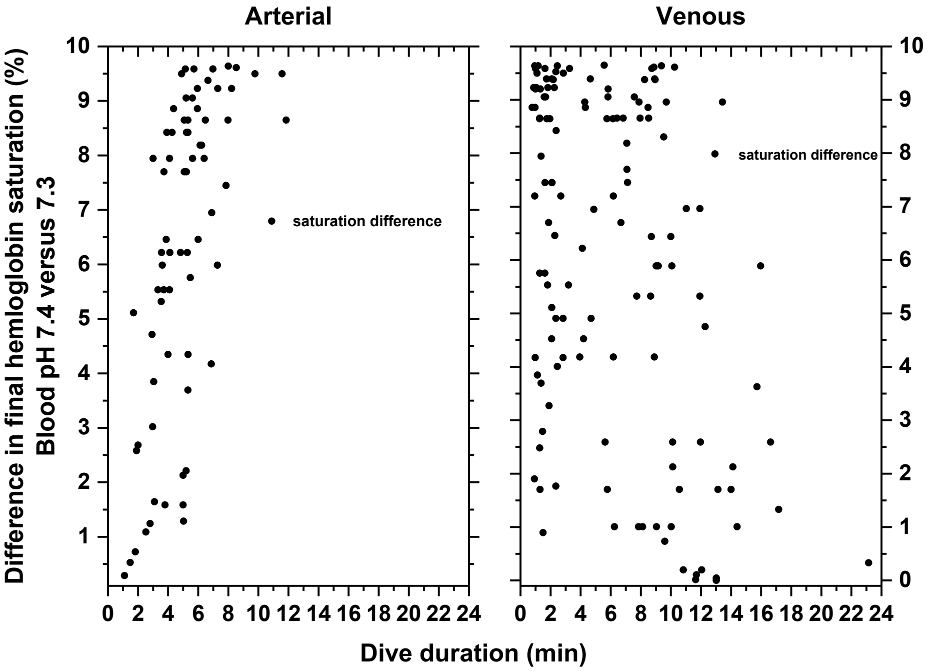

Fig. S1. Difference in final arterial and venous Hb saturations calculated at pHs 7.4 and 7.3 versus dive duration.

Journal of Experimental Biology • Supplementary information

Table S1. Blood gas, pH, and lactate analyses of blood samples with lactate concentration ([lactate] < 1.5 mM) in emperor penguins. Samples collected opportunistically from indwelling catheters during anesthesia, during dives, and at rest during isolated dive hole studies in 2004-2008 (Ponganis et al., 2009; Ponganis et al., 2007). Hypoventilation during anesthesia provided samples with elevated P_{CO2} and [lactate] < 1.5 mM. Analyses performed with a Series 200 i-STAT portable blood gas analyzer (Abbott Point of Care Inc., East Windsor, NJ, USA) as described in those studies. Abbreviations: ID – bird identification number, BM – body mass in kilograms (kg), Site - A, arterial, V, venous, P_{CO2} - partial pressure of carbon dioxide, P_{O2} – partial pressure of oxygen, mm Hg – millimeters of mercury, mM – millimolar.

Year	ID	BM (kg)	Site	pH	P_{CO2} (mm Hg)	P_{O2} (mm Hg)	[lactate] (mM)	Comments
2005	32	31.9	A	7.30	80	289	< 0.3	Anesthesia
2005	33	22.3	A	7.38	62	287	<0.3	Anesthesia
2005	34	23.0	V	7.30	75	69	0.61	Anesthesia
2005	34	23.0	V	7.26	97	87	0.64	Anesthesia
2005	34	23.0	V	7.26	97	87	0.64	Anesthesia
2005	36	27.9	V	7.49	43	56	0.56	Anesthesia
2005	37	29.0	A	7.35	81	273	< 0.3	Anesthesia
2005	40	23.7	V	7.27	81	89	0.43	Anesthesia
2005	41	25.9	V	7.41	62	80	0.6	Anesthesia
2005	42	27.0	A	7.34	74	380	0.32	Anesthesia
2007	4	26.1	A	7.21	129	247	< 0.3	Anesthesia
2008	3	23.1	A	7.28	93	368	0.5	Anesthesia
2008	3	23.1	A	7.35	76	424	<0.3	Anesthesia
2004	21	22.9	A	7.35	53	81	0.51	Intradive – blood sampler
2004	21	22.9	A	7.35	52	89	0.75	Intradive – blood sampler
2004	21	22.9	A	7.42	44	72	1.00	Intradive – blood sampler
2004	22	24.5	V	7.30	71	31	1.31	Intradive – blood sampler
2004	29	28.2	V	7.47	48	58	1.08	Intradive – blood sampler
2004	29	28.2	V	7.47	48	58	1.03	Intradive – blood sampler
2005	31	28.1	V	7.37	62	39	0.57	Intradive – blood sampler
2004	22	24.5	V	7.45	41	52	<0.3	At rest
2005	31	28.1	V	7.49	55	30	0.65	At rest
2005	32	31.1	A	7.53	38	74	1.20	At rest
2005	34	23.0	V	7.47	51	44	1.08	At rest
2005	36	27.9	V	7.47	43	56	0.47	At rest
2005	41	25.9	V	7.53	48	41	0.59	At rest
2005	42	27.0	A	7.50	48	49	0.90	At rest
2005	44	25.1	V	7.54	49	29	0.82	At rest
2005	45	23.0	V	7.51	52	36	1.09	At rest
2005	46	27.2	V	7.56	50	39	0.46	At rest
2005	47	25.0	V	7.53	48	41	0.59	At rest
2005	48	25.2	A	7.48	43	61	0.91	At rest

Table S2. Blood gas, pH, and lactate analyses of blood samples with elevated lactate concentration ([lactate] >2 mM) in emperor penguins. Samples collected opportunistically from indwelling catheters during anesthesia, during transport, and during restraint for deployment/removal of a backpack blood sampler during isolated dive hole studies in 2004-2005 (Ponganis et al., 2009; Ponganis et al., 2007). Analyses performed with a Series 200 i-STAT portable blood gas analyzer (Abbott Point of Care Inc., East Windsor, NJ, USA) as described in those studies. Abbreviations: ID – bird identification number, BM – body mass in kilograms (kg), Site - A, arterial, V, venous, P_{CO2} - partial pressure of carbon dioxide, P_{O2} – partial pressure of oxygen, mm Hg – millimeters of mercury, mM – millimolar.

Year	ID	BM (kg)	Site	pH	P_{CO2} (mm Hg)	P_{O2} (mm Hg)	[lactate] (mM)	Comments
2004	21	22.9	A	7.43	43	67	2.34	Restraint,7 min post 3.3-min dive
2004	21	22.9	A	7.46	46	54	2.46	Pre-dive restraint
2004	21	22.9	A	7.43	48	67	2.37	Pre-dive restraint
2004	21	22.9	A	7.39	43	60	7.32	Pre-dive restraint
2004	22	24.5	V	7.31	37	49	17.03	Restraint, 10 min post 17.3-min dive
2004	29	28.2	V	7.37	62	32	9.87	Pre-dive restraint, struggle
2004	29	28.2	V	7.39	56	31	5.01	Pre-dive restraint
2005	36	27.9	V	7.29	38	81	15.41	Restraint, 10.7 min post 10.6-min dive
2005	40	23.7	V	7.33	49	50	10.70	Pre-dive restraint
2005	40	23.7	V	7.38	52	47	6.31	Restraint, post 6.6-min dive
2005	43	24.5	V	7.25	85	89	3.06	Anesthesia
2005	44	25.0	V	7.47	45	49	2.8	At rest in transport box
2005	44	25.0	V	7.23	72	52	3.08	Anesthesia
2005	48	25.0	V	7.35	52	42	5.42	Agitated in transport box

Journal of Experimental Biology • Supplementary information

Table S3. Final arterial and venous Hb saturations (calculated at pH 7.40 and pH 7.30), initial arterial and venous O_2 stores, respective sizes of the remaining blood O_2 stores at end of the dive, and net O_2 contributions of the respective blood O_2 stores to diving metabolic rate (DMR) for dives of five to six-min duration. Initial O_2 stores were calculated with pH 7.5 Hb saturations. Final saturations were calculated with O_2-Hb saturation curves at two pH values due to uncertainty of final blood pH for dives of this duration. At the end of the dive, mean arterial and venous O_2 stores yielded a total blood O_2 store of 12.5 ml O_2 kg^{-1} and 10.7 ml O_2 kg^{-1}, respectively, for pH 7.4 and 7.3. The arterial store had decreased 24% and 31% for pH 7.4 and 7.3, while the venous store declined 53% and 61%, respectively from start-of-dive values. The combined arterial and venous contribution to DMR was 1.6 and 1.8 ml O_2 kg^{-1} min^{-1}, respectively for pH 7.4 and 7.3. For these five to six-min dives, the blood contributions to DMR for the pH 7.4 and 7.3 results were 145% and 164% of that previously estimated with end-of-dive saturations calculated at pH 7.4 for dives of all durations in the study (Meir and Ponganis, 2009; Williams et al., 2011). Data in these selected groups of dive durations were normally distributed (Kolmogorov-Smirnov) and expressed as mean and standard error (SE). Abbreviations: Max – maximum, Min – minimum.

Arterial O_2 Store - Hb saturation calculated at pH 7.4, n = 22						
	Dive Duration	Initial Arterial O_2 Store	Final Hb Saturation (pH 7.4)	Final Arterial O_2 Store	% O_2 Store Remaining	Contribution to DMR
	min	ml O_2 kg^{-1}	%	ml O_2 kg^{-1}	%	ml O_2 kg^{-1} min^{-1}
Mean	5.4	7.9	74	6.1	76.2	0.4
SE	0.07	0.02	2.8	0.23	2.89	0.05
Max	6.0	8.1	96	7.8	97.9	0.8
Min	5.0	7.8	55	4.4	56.0	0.1
Median	5.3	7.9	72	5.8	73.6	0.5
Venous O_2 Store – Hb saturation calculated at pH 7.4, n=6						
	Dive Duration	Initial Venous O_2 Store	Final Hb Saturation (pH 7.4)	Final Venous O_2 Store	% O_2 Store Remaining	Contribution to DMR
	min	ml O_2 kg^{-1}	%	ml O_2 kg^{-1}	%	ml O_2 kg^{-1} min^{-1}
Mean	5.7	13.1	38	6.4	47.2	1.2
SE	0.04	0.74	11.0	1.81	13.0	0.27
Max	5.8	14.9	66	10.9	76.4	2.1
Min	5.6	10.8	5	0.8	5.8	0.5
Arterial O_2 Store - Hb saturation calculated at pH 7.3, n = 22						
	Dive Duration	Initial Arterial O_2 Store	Final Hb Saturation (pH 7.3)	Final Arterial O_2 Store	% O_2 Store Remaining	Contribution to DMR
	min	ml O_2 kg^{-1}	%	ml O_2 kg^{-1}	%	ml O_2 kg^{-1} min^{-1}
Mean	5.4	7.9	68	5.5	69.4	0.4
SE	0.07	0.02	3.4	0.28	3.49	0.05
Max	6.0	8.1	95	7.7	96.2	0.7
Min	5.0	7.8	45	3.7	46.2	0.0
Venous O_2 Store – Hb saturation calculated at pH 7.3, n = 6						
	Dive Duration	Initial Venous O_2 Store	Final Hb Saturation (pH 7.3)	Final Venous O_2 Store	% O_2 Store Remaining	Contribution to DMR
	min	ml O_2 kg^{-1}	%	ml O_2 kg^{-1}	%	ml O_2 kg^{-1} min^{-1}
Mean	5.7	13.1	32	5.2	38.7	1.4
SE	0.04	0.74	9.7	1.59	11.35	0.23
Max	5.8	14.9	58	9.5	63.8	2.2
Min	5.6	10.8	3	0.5	3.7	0.7

Journal of Experimental Biology • Supplementary information

Table S4. Final arterial and venous Hb saturations (calculated at pH 7.3), respective sizes of the remaining blood O_2 stores at end of the dive, and net O_2 contributions of the respective blood O_2 stores to diving metabolic rate (DMR) for dives of 10 to 12-min duration. Initial O_2 stores were calculated with pH 7.5 Hb saturations. Final saturations were calculated with the O_2-Hb saturation curve at pH 7.3. At the end of the dive, mean arterial and venous O_2 stores yielded a total blood O_2 store of 5.6 ml O_2 kg^{-1}. The arterial store had decreased 48% while the venous store declined 87% from start-of-dive values. The blood O_2 store contribution to DMR, estimated with Hb saturations calculated at pH 7.3 during a 10-min dive was 1.4 ml O_2 kg^{-1} min^{-1}, 127% of that previously calculated at pH 7.4 with mean values of all dives (Williams et al., 2011). Abbreviations: Max – maximum, Min – minimum.

Arterial O_2 Store, n = 3						
	Dive Duration	Initial Arterial O_2 Store	Final Hb Saturation (pH 7.3)	Final Arterial O_2 Store	% O_2 Store Remaining	Contribution to DMR
	min	ml O_2 kg^{-1}	%	ml O_2 kg^{-1}	%	ml O_2 kg^{-1} min^{-1}
Mean	11.1	8.0	51	4.1	51.6	0.4
SE	0.65	0.04	3.4	0.28	3.77	0.05
Max	11.9	8.0	58	4.7	59.1	0.4
Min	9.8	7.9	47	3.8	47.8	0.3
Venous O_2 Store, n = 14						
	Dive Duration	Initial Venous O_2 Store	Final Hb Saturation (pH 7.3)	Final Venous O_2 Store	% O_2 Store Remaining	Contribution to DMR
	min	ml O_2 kg^{-1}	%	ml O_2 kg^{-1}	%	ml O_2 kg^{-1} min^{-1}
Mean	10.9	11.9	9	1.5	12.7	1.0
SE	0.22	0.51	2.8	0.46	4.31	0.06
Max	12.0	15.2	38	6.2	61.9	1.3
Min	10.0	9.4	0	0	0.0	0.4

Journal of Experimental Biology • Supplementary information

Table S5. Contribution of post-dive resynthesis of phosphocreatine (PC) in pectoralis-supracoracoideus muscle complex to body O_2 consumption in emperor penguins. Based on 25 kg body mass, pectoralis-supracoracoideus (PEC/SC) muscle mass = 25% body mass (Ponganis et al., 1997), estimated PC depletion during six-min and 10-min dives (Williams et al., 2012), 0.268 mmol ATP ml^{-1} O_2, one ATP equivalent to one PC (Williams et al., 2012), and restoration of PC within two min (McMahon and Jenkins, 2002; Yoshida et al., 2013). Abbreviation: ATP – adenosine triphosphate.

		6-min dive		10-min dive
PC depletion (mmol kg^{-1} muscle)		10		15
PC depletion in Pec/SC muscle (mmol)		63		94
Body O_2 consumption for resynthesis in 2 min (ml O_2 kg^{-1} min^{-1})		5		7

References

McMahon, S. and Jenkins, D. (2002). Factors Affecting the Rate of Phosphocreatine Resynthesis Following Intense Exercise. *Sports Medicine* **32**, 761–784.

Meir, J. U. and Ponganis, P. J. (2009). High-affinity hemoglobin and blood oxygen saturation in diving emperor penguins. *Journal of Experimental Biology* **212**, 3330–3338.

Ponganis, P. J., Costello, M. L., Starke, L. N., Mathieu-Costello, O. and Kooyman, G. L. (1997). Structural and biochemical characteristics of locomotory muscles of emperor penguins, *Aptenodytes forsteri*. *Respiration Physiology* **109**, 73–80.

Ponganis, P. J., Stockard, T. K., Meir, J. U., Williams, C. L., Ponganis, K. V. and Howard, R. (2009). O_2 store management in diving emperor penguins. *Journal of Experimental Biology* **212**, 217–224.

Ponganis, P. J., Stockard, T. K., Meir, J. U., Williams, C. L., Ponganis, K. V., van Dam, R. P. and Howard, R. (2007). Returning on empty: extreme blood O_2 depletion underlies dive capacity of emperor penguins. *Journal of Experimental Biology* **210**, 4279–4285.

Williams, C. L., Meir, J. U. and Ponganis, P. J. (2011). What triggers the aerobic dive limit? Muscle oxygen depletion during dives of emperor penguins. *Journal of Experimental Biology* **214**, 1801–1812.

Williams, C. L., Sato, K., Shiomi, K. and Ponganis, P. J. (2012). Muscle energy stores and stroke rates of emperor penguins: implications for muscle metabolism and dive performance. *Physiological and Biochemical Zoology* **85**, 120–133.

Yoshida, T., Abe, D. and Fukuoka, Y. (2013). Phosphocreatine resynthesis during recovery in different muscles of the exercising leg by 31P-MRS. *Scandinavian Journal of Medicine & Science in Sports* **23**, e313–e319.

Journal of Experimental Biology • Supplementary information
